# Role of reimbursement and Physicians' awareness in the survival of sorafenib‐eligible advanced hepatocellular carcinoma patients

**DOI:** 10.1002/kjm2.12838

**Published:** 2024-05-02

**Authors:** Hui‐Ling Huang, Te‐Sheng Chang, Lariza Marie Canseco, Fan Wu, Sheng‐Nan Lu

**Affiliations:** ^1^ Department of Nursing Chang Gung Memorial Hospital Chiayi Taiwan; ^2^ Department of Management Information Systems National Chung Cheng University Chiayi Chiayi Taiwan; ^3^ Department of Hepatology and Gastroenterology, Division of Internal Medicine Chang Gung Memorial Hospital Chiayi Taiwan; ^4^ College of Medicine Chang Gung University Taoyuan Taiwan; ^5^ Section of Gastroenterology, Department of Internal Medicine De Los Santos Medical Center Quezon City Philippines; ^6^ Department of Hepato‐Gastroenterology, Division of Internal Medicine Kaohsiung Chang Gung Memorial Hospital Kaohsiung Taiwan

**Keywords:** clinical effectiveness, hepatocellular carcinoma, pharmacological efficacy, reimbursement, sorafenib

## Abstract

In 2008, sorafenib became the first approved systemic therapeutic agent for advanced HCC. Although its pharmacological efficacy has been established, reimbursement for such a new, high‐cost drug, as well as physicians' awareness and prescription practice, likewise contribute to its clinical effectiveness. We therefore conducted a retrospective study using 38 sorafenib‐eligible, advanced HCC patients when sorafenib was approved but not yet reimbursed as a control and 216 patients during the reimbursed era. Study group showed longer survival at 8.2 months versus the control's 4.9 months (*p* = 0.0063 hazard ratio: 0.612 [0.431 ~ 0.868], *p* = 0.0059). Among the 42 (19.4%) patients who survived more than 2 years, 50% had tumor rupture, and all 32 patients with portal vein tumor thrombus and/or extrahepatic metastasis received sorafenib (*p* = 0.003). Furthermore, during their first 2 years of HCC management, sorafenib had been given in 29.1% of the treatment courses among survivors between 2 and 5 years while it was prescribed in 55.8% among the more than 5 years survivor group (*p* < 0.001). In conclusion, survival of sorafenib‐eligible HCC patients significantly improved after reimbursement. Patients who underwent longer sorafenib treatment had a survival advantage, except for those with tumor rupture. Reimbursement and awareness of prescriptions for a newly introduced medication therefore improve clinical effectiveness.

## 1 INTRODUCTION

Hepatocellular carcinoma (HCC) poses a significant global public health challenge. Currently, it ranks as the sixth most common cancer worldwide and is the third leading cause of cancer‐related deaths.^1^, ^2^, ^3^ In recent decades, the overall survival rates for HCC in Taiwan have shown continuous improvement.^4^, ^5^, ^6^ Unfortunately for some patients, they are already in advanced stages at the time of diagnosis, and systemic therapy becomes the primary treatment option.^7^, ^8^, ^9^, ^10^, ^11^ In the past, there was a lack of evidence supporting the use of systemic therapy to improve the survival rates of advanced HCC patients.^7^ However, in 2008, the phase III Sorafenib Hepatocellular Carcinoma Assessment Randomized Protocol (SHARP) trial was successful; this marked a significant milestone for the approval of sorafenib as the first systemic therapy for advanced HCC by the United States Food and Drug Administration (FDA).^12^, ^13^, ^14^ In April 2010, it gained recognition from the Taiwan Food and Drug Administration (TFDA) and was subsequently officially included in Taiwan's National Health Insurance system in August 2012.

Research has demonstrated that healthcare insurance reimbursement policies are pivotal in ensuring access to healthcare and medications, ultimately leading to enhanced survival rates in cancer patients.^5^, ^15^, ^16^, ^17^, ^18^, ^19^ Taiwan boasts a comprehensive national healthcare system, the National Health Insurance (NHI), characterized by a mandatory single‐payer mechanism for healthcare fund distribution. This system promises equal access to medical services for all citizens and currently maintains an impressive coverage rate of 99.9%.^18^, ^19^ However, it is worth noting that before August 2012, advanced liver cancer patients in Taiwan did not benefit from healthcare insurance coverage for systemic therapy medications.

Since its approval in 2008, numerous studies have investigated sorafenib's pharmacological mechanisms and survival outcomes.^20^, ^21^ However, to date, there has been relatively limited literature discussing the impact of national healthcare policies, particularly national health insurance, on survival outcomes. In this study, we examined the survival benefits of individuals receiving reimbursed treatment with sorafenib from the perspective of national healthcare policies. We also explored the cognitive aspects and prescription practices related to sorafenib. We hope that the results of this study will provide valuable insights for healthcare policies and clinical prescription practices.

## 2 MATERIALS AND METHODS

### 2.1 Patients and methods

Data regarding patients with HCC coded as ICD‐9155 and ICD‐10 C220 were extracted from the cancer registry of a regional hospital in Taiwan. Sorafenib obtained approval in April 2010 from the TFDA. Thus, the present study recruited advanced HCC patients between April 2010 and May 2019 for clinical data analysis. The analysis encompasses two distinct time periods: a control group (April 1, 2010 to July 31, 2012), prior to the NHI's reimbursement of sorafenib, and a study group (August 1, 2012, to May 30, 2019) from the initiation of first‐line sorafenib reimbursement by the NHI until the reimbursement of second‐line regorafenib.

The inclusion criteria used in this study to become sorafenib treatment‐eligible are as follows: Barcelona Clinic Liver Cancer classification (BCLC) stage C, Child‐Pugh class A liver function reserve, and no initial surgical resection or local ablation therapy. We reviewed the cancer registration database of the regional hospital and obtained data on age, gender, viral etiology, Child‐Pugh classification, Albumin‐Bilirubin (ALBI) grade, BCLC staging, American Joint Committee on Cancer (AJCC) staging, tumor characteristics, diagnostic data, diagnosis date, initial treatment modality and medication history of sorafenib. Survival status was gathered from the national mortality database, and the information on liver cancer survival was calculated and analyzed by merging these two databases. Additionally, the medical records of patients who survived for 2 years or longer were more extensively examined. This study was approved by the Institutional Review Board of Chang Gung Memorial Hospital (IRB No: 202300271B0).

### 2.2 Statistical analysis

Quantitative variables were presented as percentages, and the chi‐square test and Fisher's exact test were employed to compare categorical variables. The overall survival disparities between groups were assessed using Kaplan–Meier survival curves and the log‐rank test. A *p*‐value <0.05 in a two‐tailed test was regarded as statistically significant. Hazard ratios (HRs) with 95% confidence interval (CI) were obtained using the Cox proportional hazard model. Additionally, swimmer plots were employed to present the treatment sequence and modalities.

## 3 RESULTS

### 3.1 Patient selection and categorization

**FIGURE 1:**
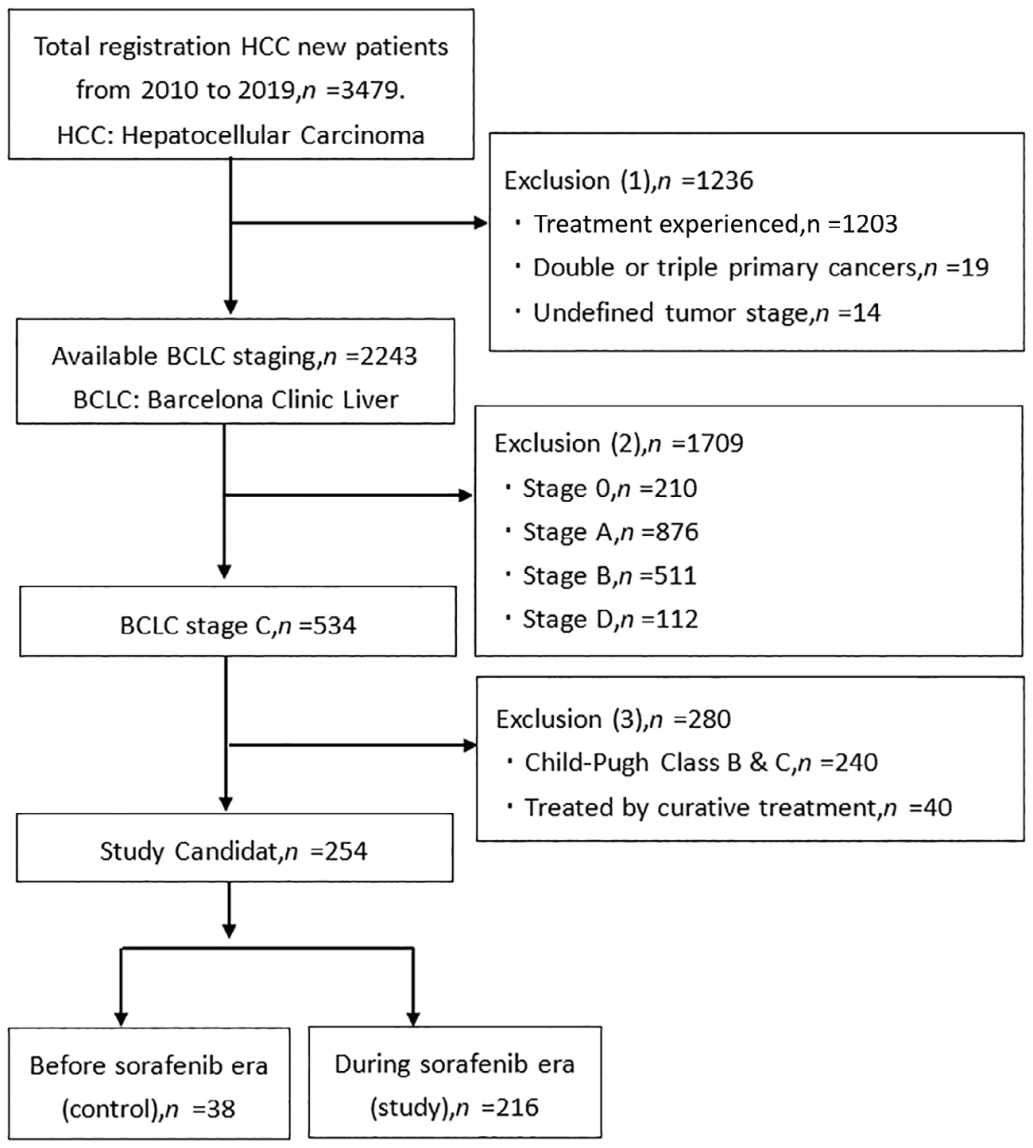
Patient enrollment flow diagram. BCLC, Barcelona clinic liver cancer; HCC, Hepatocellular carcinoma.

A total of 3479 HCC patients were enrolled from 2010 to 2019. Ultimately, a total of 254 HCC patients were included in this study according to the eligibility criteria. Based on the dates of NHI reimbursements, they were further categorized into two groups. The control group consisted of 38 patients diagnosed with liver cancer during the period when sorafenib was not yet reimbursed by the NHI, while 216 patients were classified as the study group during the era of sorafenib reimbursement, as shown in Figure 1.

### 3.2 Clinical and treatment features of the study population

Table 1 presents the baseline characteristics of 254 HCC patients in our study population. The majority of patients in both the control and study groups were males and were classified as AJCC stage IV. The control group had significantly younger age (60.7 vs. 65.0 years, *p* = 0.006), while the study group had lower proportion of ALBI grade II (69.9% vs. 94.7%, *p* = 0.001), lower proportion of supportive treatment (5.6% vs. 23.7%, *p* < 0.001), and higher proportion of sorafenib use (76.4% vs. 13.2%, *p* < 0.001). A total of 170 patients were treated with sorafenib, including 5 in the control group and 165 in the study group. Most of them were prescribed within 6 months after diagnosis (92.4%). Majority underwent sorafenib treatment for less than 6 months (78.6%) and discontinued the drug primarily due to disease progression including death (68.2%). Since only five controls were treated with sorafenib, no inter‐groups comparison was made.

**TABLE 1:** Clinical and treatment features of the advanced HCC patients.

Variables	Control (*n =* 38)	Study(*n =* 216)	*p* value
	*n* (%)	*n* (%)	
Gender			0.373
Male	29 (76.3%)	178 (82.4%)	
Female	9 (23.7%)	38 (17.6%)	
Age, years (mean, SD)	60.7, 11.4	65.0, 12.9	0.006*
Viral etiology			0.143
HBsAg(+), anti‐HCV(−)	19 (50%)	79 (36.6%)	
HBsAg(−), anti‐HCV(+)	10 (26.3%)	86 (39.8%)	
HBsAg(+), anti‐HCV(+)	6 (15.7%)	20 (9.3%)	
HBsAg(−), anti‐HCV (−)	3 (7.9%)	31 (14.4%)	
Alpha‐Fetoprotein, AFP (ng/mL)			0.737
<400	21 (55.3%)	113 (52.3%)	
>400	17 (44.7%)	103 (47.7%)	
ALBI grade			0.001*
I	2 (5.3%)	65 (30.1%)	
II	36 (94.7%)	151 (69.9%)	
AJCC stage			0.946
III	15 (39.5%)	84 (38.9%)	
IV	23 (60.5%)	132 (61.1%)	
Tumor location			0.950
Right lobe	17 (44.7%)	98 (45.4%)	
Left lobe	3 (7.9%)	14 (6.5%)	
Both lobes	18 (47.4%)	104 (48.1%)	
Tumor number			0.617
1	9 (23.7%)	53 (24.5%)	
2 or 3	8 (21.1%)	32 (14.8%)	
>3	21 (55.3%)	131 (60.6%)	
Largest tumor diameter (cm)			0.344
≤5	10 (26.3%)	43 (19.9%)	
5–10	14 (36.8%)	107 (49.5%)	
>10	14 (36.8%)	66 (30.6%)	
Portal vein thrombosis, PVT			0.931
NO	14 (36.8%)	78 (36.1%)	
YES	24 (63.2%)	138 (63.9%)	
Lymph node metastasis			0.719
NO	26 (68.4%)	154 (71.3%)	
YES	12 (31.6%)	62 (28.7%)	
Distant metastasis			0.446
NO	22 (57.9%)	139 (64.4%)	
YES	16 (42.1%)	77 (35.6%)	
Tumor rupture			0.662
NO	35 (92.1%)	194 (89.8%)	
YES	3 (7.9%)	22 (10.2%)	
Initial treatment modality			0.000*
Locoregional therapy	13 (34.2%)	33 (15.3%)	
Systemic therapy	11 (28.9%)	80 (37.0%)	
Combination therapy	5 (13.2%)	91 (42.1%)	
Supportive treatment	9 (23.7%)	12 (5.6%)	
Initial prescription of sorafenib			0.000*
NO	33 (86.8%)	51 (23.6%)	
<6 months after diagnosis	5 (13.2%)	152 (70.4%)	
>6 months after diagnosis	0	13 (6%)	
Duration of Sorafenib use			0.000*
NO	33 (86.8%)	51 (23.6%)	
<6 months	3 (7.9%)	130 (60.2%)	
>6 months	2 (5.3%)	35 (16.2%)	
Reason for sorafenib withdrawal			0.951
Disease progression	2 (40%)	86 (52.1%)	
Advanced to Child‐Pugh B	1 (20%)	30 (18.2%)	
Death	1 (20%)	27 (16.4%)	
Intolerance of side effects	1 (20%)	22 (13.3%)	

*Note*: Combination therapy: Simultaneous use of systemic and locoregional therapy.

Table 2 shows the hazard ratios (HR) and 95% confidence intervals (CI) of prognostic factors for overall survival by Cox proportional hazards model. In the univariate analysis, the study group (HR 0.63, CI 0.44–0.89, *p* = 0.010), prescription of sorafenib within 6 months after diagnosis (HR 0.73, CI 0.56–0.96, *p* = 0.023), and duration of sorafenib use more than 6 months (HR 0.62, CI 0.42–0.93, *p* = 0.021) were all positively associated with good overall survival. However, supportive treatment as the initial treatment modality (HR 2.04, CI 1.21–3.45, *p* = 0.008) was correlated with poor prognosis in both univariate and multivariate analyses. Thus, this indicates that absence of, delay, and insufficient sorafenib treatment duration resulted in poor overall survival. In the multivariate analysis, only supportive treatment was found to be statistically significant.

**TABLE 2:** Hazard Ratios (HR) and their 95% Confidence Interval (CI) for Overall Survival by Univariate and Multivariate Analyses by Cox Proportional Hazards Model.

Variables	Univariate analysis	*p*‐value	Multivariate analysis
	HR (95% CI)		HR (95% CI)
Groups			
Control	Reference		
Study	0.63 (0.44, 0.89)	0.010*	
Gender			
Male	1.30 (0.94, 1.79)	0.117	
Female	Reference		
Age, years (per year)	1.00 (0.99, 1.01)	0.692	
Viral etiology			
HBsAg(+), anti‐HCV(−)	0.97 (0.65, 1.44)	0.884	
HBsAg(−), anti‐HCV(+)	0.75 (0.50, 1.11)	0.153	
HBsAg(+), anti‐HCV(+)	0.95 (0.56, 1.58)	0.830	
HBsAg(−), anti‐HCV(−)	Reference		
Alpha‐fetoprotein (ng/mL)			
<400	Reference		
>400	0.86 (0.67, 1.10)	0.236	
ALBI grade			
I	Reference		
II	1.15 (0.87, 1.53)	0.335	
AJCC stage			
III	Reference		
IV	0.85 (0.66, 1.10)	0.230	
Tumor location			
Right lobes	Reference		
Left lobes	1.36 (0.82, 2.27)	0.238	
Both lobes	1.01 (0.78, 1.31)	0.949	
Tumor number			
1	Reference		
2 or 3	0.79 (0.53, 1.18)	0.248	
>3	0.84 (0.62, 1.13)	0.255	
Largest tumor diameter (cm)			
≤ 5	Reference		
5–10	1.03 (0.74, 1.43)	0.875	
> 10	1.11 (0.78, 1.58)	0.554	
Portal vein thrombosis, PVT			
NO	Reference		
YES	1.11 (0.85, 1.44)	0.448	
Lymph node metastasis			
NO	Reference		
YES	0.82 (0.62, 1.08)	0.150	
Distant metastasis			
NO	Reference		
YES	0.95 (0.73, 1.23)	0.705	
Tumor rupture			
NO	Reference		
YES	0.88 (0.58, 1.32)	0.529	
Initial treatment modality			
Locoregional therapy	Reference		Reference
Systemic therapy	0.87 (0.61, 1.25)	0.450	0.87 (0.61, 1.25)
Combination therapy	0.94 (0.66, 1.34)	0.744	0.94 (0.66, 1.34)
Supportive treatment	2.04 (1.21, 3.45)	0.008*	2.04 (1.21, 3.45)
Initial prescription of sorafenib			
NO	Reference		
<6 months after diagnosis	0.73 (0.56, 0.96)	0.023*	
>6 months after diagnosis	1.22 (0.68, 2.19)	0.510	
NO	Reference		
<6 months	0.80 (0.61, 1.05)	0.105	
>6 months	0.62 (0.42, 0.93)	0.021*	

*Note*: Using stepwise Cox regression.

### 3.3 Survival outcome of the study cohort

**FIGURE 2:**
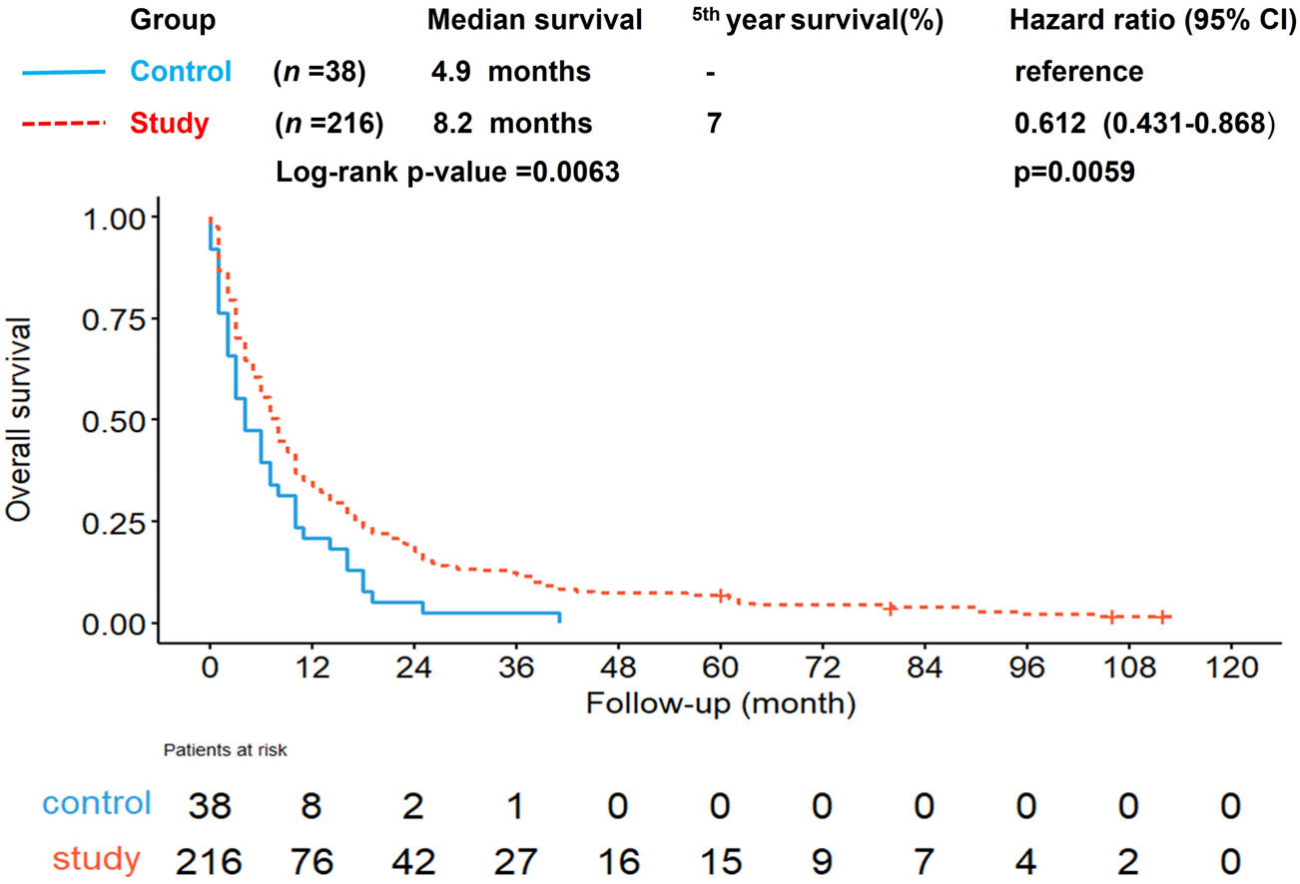
Kaplan–Meier analysis of overall survival between the control and study groups showing that 216 patients during the era of sorafenib reimbursement (study group) had a higher OS at 8.2 months (red dashed line) compared to the control (blue line) (*p* = 0.0063).

As shown in Figure 2, the study group exhibited a significantly improved overall survival (OS) of 8.2 months compared to the control group's 4.9 months, as indicated by a *p*‐value of 0.0063. The study group also had a computed 5‐year survival rate of 7% (HR 0.612, 95% CI: 0.431–0.868).

### 3.4 Proportion and duration of sorafenib use in the treatment course of patients who survived for 2 years or longer

**FIGURE 3:**
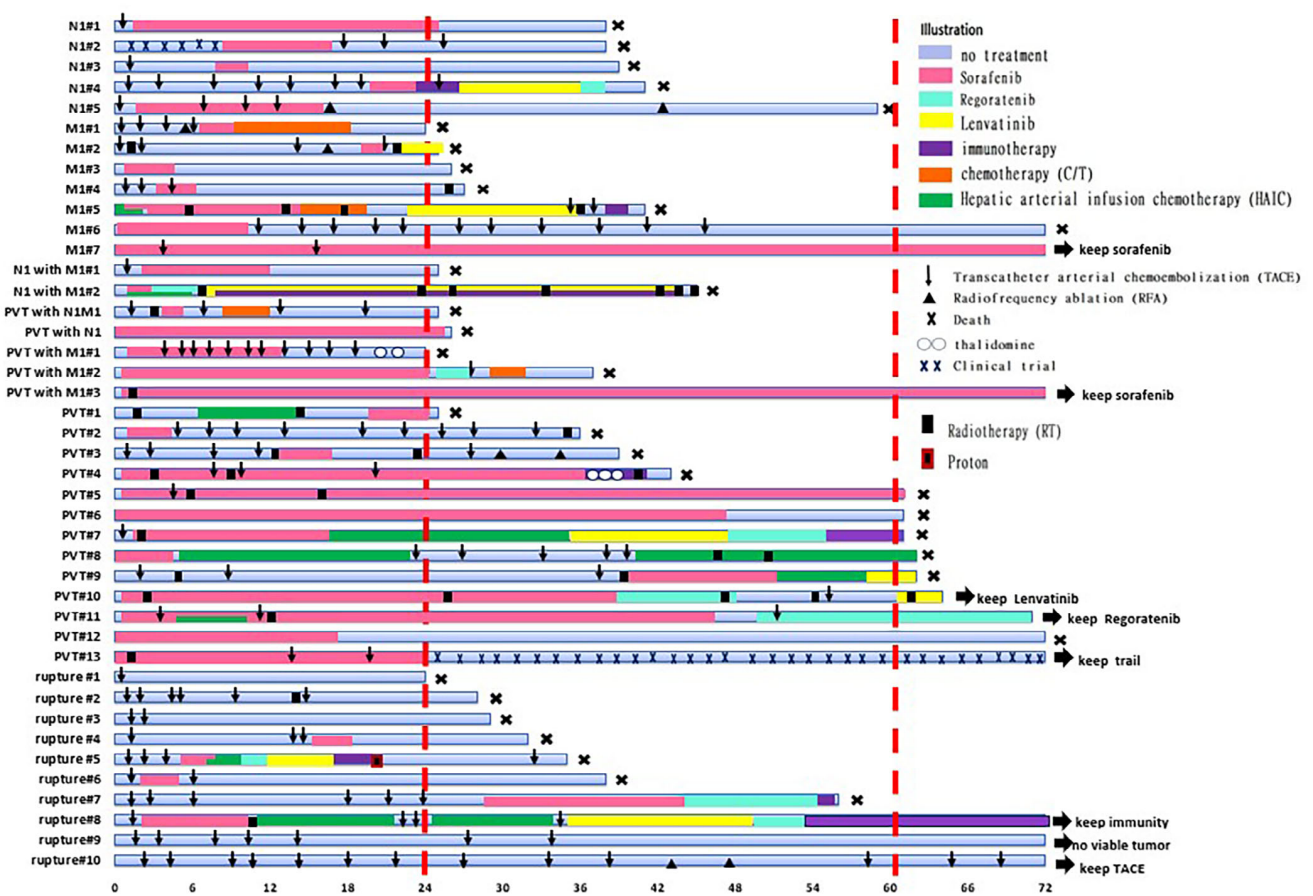
Swimmer plot presenting the different treatment modalities undergone by the 42 advanced HCC patients who survived for more than 2 years, including 15 who survived longer than 5 years. The patients were classified as BCLC stage C due to either having portal vein tumor thrombosis (PVT), extrahepatic spread (EHS), tumor rupture, or their combinations. Five out of the 10 patients with tumor rupture and all of the other 32 patients underwent sorafenib treatment.

Forty‐two patients survived for 2 years or longer, including 15 who survived for more than 5 years. The treatment courses of the 42 patients are shown by the swimmer plot in Figure 3. The patients were classified as BCLC stage C due to having portal vein tumor thrombosis (PVTT), extrahepatic spread (EHS), tumor rupture, or their combinations. Overall, 5 out of the 10 patients with tumor rupture and all of the other 32 patients underwent sorafenib treatment (*p* = 0.0003).

The following analysis was conducted in two ways, that is, inclusion or exclusion of patients with tumor rupture. Focusing on the 15 patients who survived for 5 years or longer, we observed 900 person‐months (15 patients × 5 years × 12 months) in their first 5 years. A total of 400 (44.4%) person‐months of 13 (86.7%) patients underwent sorafenib treatment. Twelve (80.0%) were prescribed within 6 months after diagnosis (Table 3, Group A1). When excluding three patients with tumor rupture, 399 (55.4%) person‐months of all 12 underwent sorafenib treatment in a 720 person‐month follow‐up. All but one (91.6%) were prescribed within 6 months after diagnosis (Table 3, Group B1). To compare the status of sorafenib use with patients who survived for 2–5 years, we shortened their observation time to 2 years. A total of 201 (55.8%) person‐months of 12 (80%) patients underwent sorafenib in a 360 person‐months follow‐up (Table 3, Group A2). When excluding three patients with tumor rupture, 201 (69.8%) person‐months of 11 (91.6%) patients underwent sorafenib treatment in a 288 person‐months follow‐up (Table 3, Group B2).

**TABLE 3:** Sorafenib treatment by person‐months of the 42 patients who survived for 2 years or longer.

Group	Survival (year)	Include tumor rupture	Observation (month)	Total number of patients	Total person‐months	Sorafenib‐treated patients	(%)	Sorafenib‐treated patients and total months	(%)	The number of patients receiving sorafenib treatment within six months of diagnosis	(%)
			A	B	C	D	E	F	G	H	I
					A × B		D/B		F/C		H/B
A1	>5	Yes	60	15	900	13	86.7	400	44.4	12	80.0
A2	>5	Yes	24	15	360	12	80.0	201	55.8	11	80.0
A3	2–5	Yes	24	27	648	23	85.1	189	29.1	15	55.5
B1	>5	No	24	12	720	12	100	399	55.4	11	91.6
B2	>5	No	24	12	288	11	91.6	201	69.8	11	91.6
B3	2–5	N0	24	20	480	20	100	178	37.1	13	65.0

*Note*: P: Fisher's exact test or Chi‐square with Yate's correction. A2 Vs A3: E (*p* = 0.6858), G (*p* < 0.001), I (*p* = 0.1211). B2 Vs B3: E (*p* = 1.0000), G (*p* < 0.001), I (*p* = 0.0331).

Overall, 27 patients survived between 2 and 5 years. A total of 189 (29.1%) person‐months of 23 (85.1%) patients had sorafenib treatment in a 648 person‐month follow‐up. Fifteen of them were prescribed within 6 months after diagnosis (Table 3, Group A3). When excluding seven patients with tumor rupture, 178 (37.1%) person‐months of 20 (100%) patients underwent sorafenib treatment in a 480 person‐months follow‐up. Thirteen of them were prescribed within 6 months after diagnosis (Table 3, Group B3).

The proportion of sorafenib treatment (80% vs. 85.1%. *p* = 0.6858) and early use of sorafenib (80% vs. 55.5%, *p* = 0.1211) were not significantly different between the patients who survived for more than 5 years compared to the 2–5 years survivor group. However, the proportion of duration of sorafenib use was significantly greater in the group who survived for more than 5 years (55.8% vs. 29.1%, *p* < 0.001). When excluding patients with tumor rupture, both the proportion of duration of sorafenib use (69.8% vs. 37.1%, *p* < 0.001) and proportion of early initiation of sorafenib (91.6% vs. 65%, *p* = 0.0331) were significantly higher in the longer survivor group compared to those who only survived between 2 and 5 years.

## 4 DISCUSSION

In the current study, we elucidated that reimbursement of sorafenib significantly improved survival among advanced HCC patients who were candidates for systemic treatment. We also noted that early and longer duration of sorafenib use resulted in increased survival. This indicates that both reimbursement and awareness of prescription can narrow the gap between pharmacological efficacy and clinical effectiveness. We therefore strongly recommend shortening the interval between drug approval and reimbursement as well as increasing education courses for newly introduced medications. The average interval from FDA approval to reimbursement by Taiwan NHI is 4 years; hence, there is still room for improvement. As shown in Table 1, around 80% of patients were treated with systemic therapy or their combinations after reimbursements for sorafenib began, compared to 42.1% prior to reimbursements. After reimbursement, the proportion of sorafenib use increased from 13.2% to 76.4%. The outcome of educational efforts is likewise valuable. We plan to monitor drugs introduced after sorafenib in the same way.

The median survival of sorafenib in two previous clinical trials was 10.7 months in the SHARP trial and 6.5 months in the Asia‐Pacific study.^12^, ^13^ When utilized as the control arm in single drug trials, the median survival was 9.9 months (Brivanib trial), 10.2 months (Sunitinib trial), 9.8 months (Linifanib), and 12.3 months (Lenvatinib).^22^ The largest global post‐marketing real‐world, GIDEON study showed a median survival of 13.6 months for Child‐Pugh class A patients, but this decreased to 10.7 months when limited to the Chinese population.^23^ The median survival of a multicentric post‐marketing single‐arm study in Taiwan was 8.6 months.^24^ All of the above studies showed the survival of patients who underwent sorafenib treatment. In other words, their results represent pharmacological efficacy. However, newly developed drugs are always expensive and doctors need time to understand these drugs, thus leading to a gap between pharmacological efficacy and clinical effectiveness. To find this gap, this present study included all candidates meeting indications for sorafenib treatment, regardless of which treatment modality the patients eventually underwent. The control subjects were from the time period in which sorafenib was approved, prior to reimbursement. Education courses introducing the drug may have been insufficient at that time. The median survival was only 4.9 months. This is similar to the control group in the Asia‐Pacific study. On the other hand, patients in the study group were from the period after sorafenib reimbursement started. Their median survival time was 8.2 months (HR 0.612). This was slightly better than that of the Asia‐Pacific study at 6.5 months (HR 0.68). Reimbursement did overcome the important barrier of sorafenib use and improved patients' survival outcomes. Another factor that can improve survival is physicians' awareness of prescriptions. Our results supported this point of view. This study presented a gap and pointed out the factors that narrowed the gap.

Based on the inclusion criteria of clinical trials, the candidates for sorafenib treatment should be Child‐Pugh class A patients and those with PVTT and EHS. The reimbursement policy of Taiwan NHI uses the same criteria.^25^ Tumor rupture is not among the criteria for reimbursement, however. In most guidelines, indications for systemic therapy are advanced HCC or BCLC stage C.^9^, ^10^, ^11^ Patients with tumor rupture are designated as having advanced HCC.^9^, ^10^, ^11^ The answer to the question of whether tumor rupture should be treated with systemic therapy remains equivocal. Only a few papers have mentioned this issue. Despite not having a detailed description in popular international guidelines, a review article has proposed an algorithm for the management of tumor rupture.^26^ Most patients are treated with surgical intervention and/or transcatheter arterial embolization. Survival among patients with tumor rupture appeared to be better compared to other groups of advanced HCC.^27^, ^28^, ^29^ Further studies should be conducted to investigate the role of systemic therapy among patients with tumor rupture. Incidence of peritoneal tumor seeding and the possible beneficial effects of systemic agents for preventing extrahepatic spread should be considered key points in this regard. In the current study, only half of patients with tumor rupture underwent sorafenib treatment. Since no conclusion can be drawn from this, we showed both scenarios of tumor rupture inclusion and exclusion in our analysis, as seen in Table 2.

Advanced HCC patients generally have quite low response rates, short disease‐free survivals, and overall survivals, not only in sorafenib treatment but in other target therapy agents for HCC as well. More than half of the patients in our study were nonresponders. Thus, the median survival was only 8.2 months indicating that more than half of the patient population died before 8.2 months of treatment. The expected survival of BCLC C patients was 11 months in the 2012 version of the EASL guidelines based on the SHARP study, and was extended to 2 years in the 2022 version based on the IMbrave 150 trial.^30^ Survival of more than 2 years should be the standard in the coming years.^11^ To visualize the individual treatment course of the 42 (19.4%) patients who survived longer than 2 years, we created the swimmer plot. This showed that majority of the responders underwent sorafenib treatment except for patients with tumor rupture. Comparing the first 2 years of treatment between subjects who survived for 2–5 years and those who survived for more than 5 years, the longer survival group had a significantly higher proportion of sorafenib treatment and initiated the said treatment earlier, within the first 6 months (*p* = 0.1211 in all patients, *p* = 0.0331 when patients with tumor rupture were excluded).

The general principle of cancer therapy is that local diseases should be treated with local therapy and systemic diseases should be treated with systemic therapy.^11^ Locoregional therapy is commonly used via detailed and skillful techniques in Eastern countries, such as Japan, Korea, and Taiwan. Good surveillance programs for high‐risk groups result in detection rates of early HCC of up to 45% or higher.^4^, ^10^ The survival rates of HCC in these countries are likewise the highest in the world.^31^ However, there is hematogenous spread for patients with advanced HCC, especially PVTT and EHS. Locoregional therapy may be effective for local lesions, but systemic therapy or combination with systemic therapy should be the treatment of choice. Shorter overall survival in the Asia‐Pacific trial and a post‐market survey in Taiwan^25^ may be due to delayed use of sorafenib. Improvements in the survival of sorafenib patients as control groups in the series of clinical trials also indicate the beneficial effects of the early use of systemic therapy. In our study group, about 80% of patients underwent sorafenib or combination treatments with sorafenib. Almost all patients who survived for 5 years used sorafenib in the first 6 months, thus emphasizing the timely use of systemic treatment for patients with advanced HCC.

Some clinical observations have shown that patients with longer survival had an increased treatment duration of sorafenib.^32^, ^33^ However, the causal relationship is hard to elucidate. In our study group, during the first 2 years of HCC management, sorafenib was only noted to be given in 29.1% of the treatment courses for the patients who survived between 2 and 5 years while it was prescribed in 55.9% of the courses of treatment among those who survived for more than 5 years (*p* < 0.001). This result strongly suggests that prolonged use of sorafenib is correlated with longer survival. But why do patients discontinue sorafenib? This drug is currently reimbursed without co‐payment in our NHI. Personal economical consideration should therefore not be an issue. Patients prescribed sorafenib are evaluated for treatment response using CT scans or MRI and for liver function reserve using blood tests every 2 months. Patients beyond stable disease as shown by imaging and those beyond Child‐Pugh class A are no longer reimbursed. This is a major reason for withdrawal from sorafenib. The other important factor is the intolerance of its adverse effects. Therefore, treatment duration also depends on treatment response and/or side effects rather than budget consideration or doctors' decision alone. As shown in Table 1, most of the patients withdrew from sorafenib due to disease progression and only 1/7 were due to intolerance of side effects. However, the median survival was only 8.2 months and 5‐year survival was only 7% in our study. The response rate to sorafenib was far from the expected values. Despite narrowing the gap between pharmacological efficacy through education and reimbursement, the treatment outcomes of sorafenib showed significant improvement but still did not meet the expected outcomes. We therefore need more effective drugs. Fortunately, several systemic therapeutic agents have already been approved. The study design and data analysis methods of this study can serve as a reference for evaluating and narrowing the gap between pharmacological efficacy and clinical effectiveness.

Furthermore, a single‐center study might experience practice bias and limited sample size especially in the control group. Based on this study, we plan to persuade our colleagues to conduct a validation survival analysis using the national database. A significant part of this study is drawing the individual treatment course using a swimmer plot. Collecting details of clinical data from medical records is time‐consuming. We drew a complicated treatment course of 42 patients in a swimmer plot. It would be difficult to extend this. An adequately sized, detailed study can enhance the value of pure big data analysis. Sorafenib is the first and the oldest systemic therapy for HCC. Is the study out of date? The study's design and point of view are novel. We discussed the gap between the pharmacological efficacy and clinical effectiveness of sorafenib in an era of single agents. It is more easily understood than a study with multiple treatment agents. In the future, we will use the same model to analyze detailed information in a regional hospital and extend it to the national database in four reimbursed stages, that is, no systemic therapy, sorafenib only, multiple first‐line and second‐line systemic drugs, and beginning an immunotherapy‐based combination.

## 5 CONCLUSIONS

In conclusion, the survival of sorafenib‐eligible HCC patients significantly improved after reimbursement began. Patients who underwent longer sorafenib treatment had a survival advantage except for those patients with tumor rupture. Reimbursement and awareness of prescription for a newly introduced medication therefore improve clinical effectiveness. This analysis model will extend to a larger database and eras of other reimbursed therapeutic agents in the future.

## CONFLICT OF INTEREST STATEMENT

The authors declare no conflict of interest.
